# Linking gut microbiome with the feeding behavior of the Arunachal macaque (*Macaca munzala*)

**DOI:** 10.1038/s41598-021-01316-0

**Published:** 2021-11-09

**Authors:** Avijit Ghosh, Mukesh Thakur, Lalit Kumar Sharma, Kailash Chandra

**Affiliations:** 1 Zoological Survey of India, M Block, New Alipore, Kolkata, West Bengal 700053 India; 2 Department of Zoology, University of Calcutta, Ballygunge, Kolkata, West Bengal 700019 India

**Keywords:** Ecology, Genetics, Microbiology, Zoology

## Abstract

Exploring the gut microbiome is an emerging tool for monitoring wildlife health and physiological conditions which often sustained under the variety of stresses and challenges. We analyzed gut microbiome of Arunachal macaque (*Macaca munzala*) of two disjunct populations from Arunachal Pradesh, India, to validate whether the geography or the feeding habits plays a principal role in shaping the gut microbiome in natural populations. We observed geography has a mere effect but feeding habits (i.e. feeding upon the leftover food and crop-raiding) significantly influenced the gut microbiome composition. The phylum Proteobacteria found to be enriched in leftover feeding group while phylum Bacteroidetes was differentially abundant in crop-raiding group. We observed predominant phyla Firmicutes followed by Proteobacteria and Bacteroidetes with the dominant classes represented by the Clostridia. Interestingly, one individual with known diarrheal/metabolic disorder exhibited complete dominance of the order Bacillales and showed 100% sequence similarity with genus *Solibacillus*. We raise concern that shift in diet of macaques may compel them to expose for various human diseases as two macaques feeding upon the leftover food exhibited dysbiotic gut microbiome. The present study provides the pragmatic evidences of how the alteration of food resources can harm the physiological condition of the macaques in wild and raises alarm to the forest officials/managers in strategise planting of natural food resources and monitor anthropogenic activities in the distribution of Arunachal macaques.

## Introduction

The composition of gut microbiota in mammals is chiefly shaped by the diet of the host, phylogeny and their varying habitats^1,2^. Function of mammalian gut microbiome ranges from digestion of complex food materials to raise defence against infectious microbes^3–5^.Though, the mechanism of food digestion is evolutionarily conserved across vertebrate taxa, the anatomy of vertebrate digestive tracts are linked with the structural and nutritional qualities of specific diets. Primates often show immense diversity in their dietary specialization, anatomy of gastrointestinal systems and gut microbiome composition^6^. Among Old World monkeys, Colobines are folivorous and bear ruminants gastro-intestine system that allows them to exploit unique feeding niches^7,8^. Their gut microbiomes have abilities to convert hard-to-digest plant structural compounds such as cellulose into short-chain fatty acids (SCFA) which are then absorbed directly by the host that supply up to 70% of their daily energy needs, help in nutrient absorption, and prevent the accumulation of toxic metabolic byproducts^3,9^. Non-human primates prefer high quality food (fruits and young leaves) and often shift their diet in winter to low-quality foods like mature leaves, gum and roots with high cellulose and dietary fiber content^10–12^. Previous studies on primates found diet and host physiology as the key factors regulating gut microbiome composition^13–15^. However, gut microbime of primates also gets influenced by miscellaneous factors such as individuals housed in different captive facilities^16,17^, anthropogenic disturbances in wild^13,18^ and different geographical origin of various populations^19–21^. A recent study on Long-tailed macaques (*Macaca fascicularis*) also reported variability among the oral and fecal microbiome between wild-born macaques that lived in the natural habitats and those reared in captive facilities.

**Figure 1:**
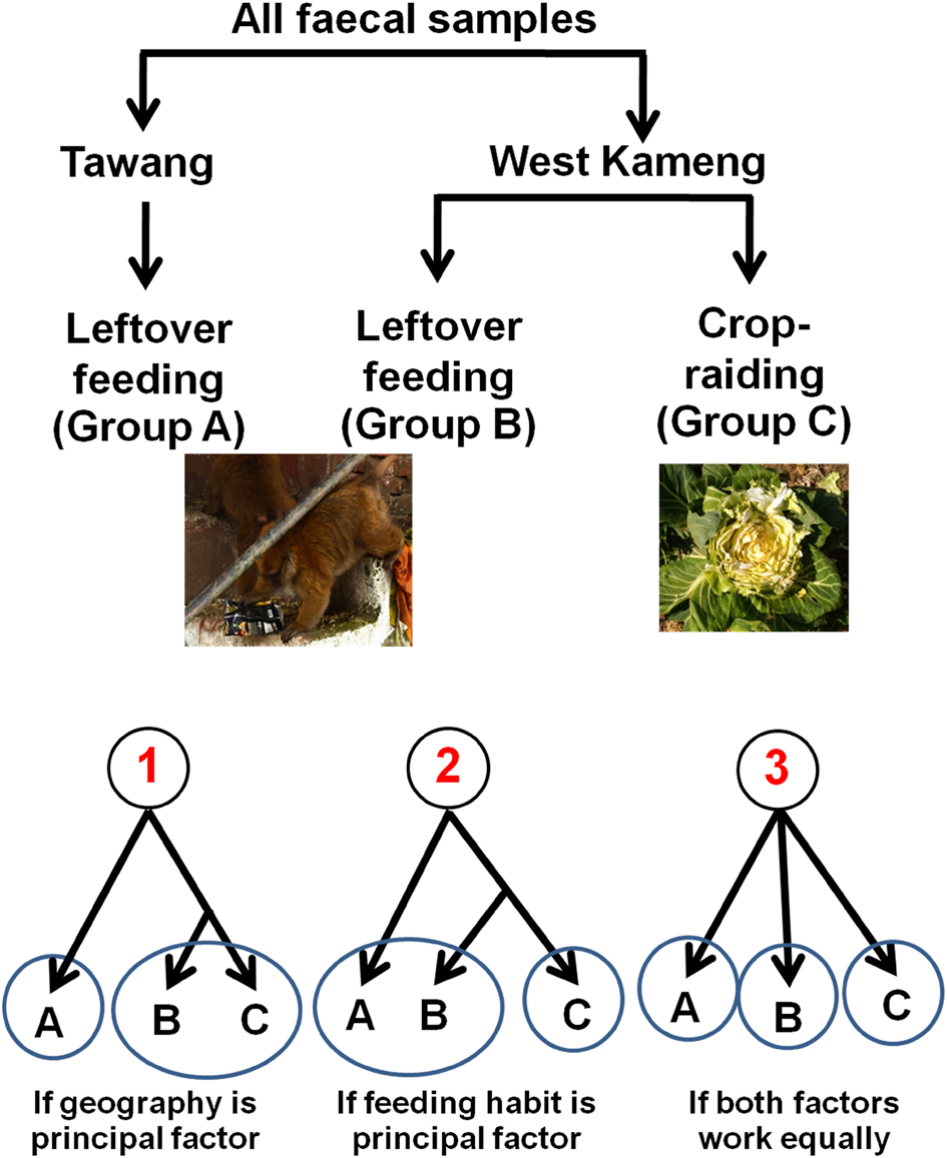
Schematic diagram of the study design and hypothesis of the present study. Three probable scenarios illustrated according to geographical origin of the samples and feeding behaviour of the study groups.

Arunachal macaque (*Macaca munzala*) is an endangered cercopithecine macaque found exclusively in temperate montane broad-leaf and conifer forests (2000–3500 m asl.) of Western Arunachal Pradesh in Northeastern India^22,23^. Earlier observations reported that diet of Arunachal macaque is predominantly consist of the pith of *Erythrina* in winter, leaves of various plant species in spring^24^ and fruits of *Elaeagnus parvifolia* in July–August^25^.We on the pilot attempts displayed an altered gut bacterial composition in Arunachal macaque which surprisingly resembled with humans indicating nature of anthropogenic food provisioning^26^. Based on our field observations of Arunachal macaques, we found two types of feeding behavior, one group encountered to feed upon the left over human food at Tawang and West Kameng, while the second group was crop-raiding and mostly encountered to feed upon the cultured vegetables at West Kameng. Thus, we testify the hypothesis which factor—the geographical origin or the feeding behavior (diet type) influence the gut microbiome of Arunachal macaques in Tawang and West Kameng (Fig. 1).

## Methods

### Sample collection

We collected ten fresh fecal samples of *M. munzala* which were defecated in front of the research team and stored immediately in 95% ethanol following Song et al.^27^. All fecal samples were collected during December 2019 and we presumed that gut microbiome might not be influenced due to the seasonal availability/variability of food resources. Based on the geographical origin, these samples represented two wild populations, Tawang (Group A of two samples) and West Kameng (Group B and C of four samples each). Two individuals (MT1398 and MT2182) from Tawang belong to two different troops often observed to feed upon the human leftover food from dumping sites. Four individuals from West Kameng (group B) belong to a resident troops living near a monastery and had similar feeding behavior like group A, while the other four individuals of group C were the crop raiding troops. In addition, we also included data from our previous study^26^ in group A. Interestingly, we also monitored a macaque from West Kameng (group B; #MT2173) that seemed to experience diarrheal/metabolic disorder (Fig. S1). We extracted DNA from the inner and outer part of the fecal samples following QIAamp DNA Stool Mini Kit (Qiagen, Germany) for identifying species, individuals and gut microbiome analysis.

### Species and individual identification

Since, other sympatric macaque species were also present in the study area, we first identified species of macaques by sequencing the partial fragment of mitochondrial d-loop^28^. Maximum likelihood (ML) phylogenetic tree was reconstructed in MEGA X^29^ with 1000 bootstrap runs using Hasegawa-Kishino-Yano model and gamma distribution of substitution rate (HKY + G) to confirm the species of origin. Although, the fecal samples were defecated in front of research team, we still genotyped each sample thrice with nine polymorphic microsatellite loci for individual identification to avoid the same sample being analyzed for gut microbiome analyses^26,30^. The obtained genotypes were analyzed to determine unique individuals using GenAlEx v6^31^ and genealogical relationship among individuals was determined using ML-RELATE^32^.

### Library preparation and amplicon sequencing

We PCR amplified V3-V4 hyper-variable region of 16S rRNA gene using Pro341F/Pro805R^33,34^ or 341F/805R primer pair^35,36^ and the 16S metagenomic sequencing library preparation was performed using standard Illumina protocol (https://support.illumina.com/downloads/16s_metagenomic_sequencing_library_preparation.html). The obtained amplicon libraries were purified using 1X AMpureXP beads and checked on Agilent DNA1000 chip using Bioanalyzer2100. The quantification of the purified library was performed in Qubit Fluorometer2.0 using Qubit dsDNA HS Assay kit and the sequencing of amplicons were performed on Illumina MiSeq (2 × 300 bp reads)/HiSeq 2500 using Rapid Run chemistry (2 × 250 bp reads).

### Data analyses

The obtained sequences were processed using Mothur MiSeq SOP pipeline for analysis of 16S rRNA sequences^37,38^. We allowed maximum of 8 homopolymers in a sequence and removed all sequences containing N. Further, the chimeric sequences were searched using the VSEARCH algorithm^39^ and the taxonomic assignment of the sequences made using Bayesian classifier with 80% bootstrap support with Silva v138.1 reference data^40^. The sequences of mitochondria and chloroplast were removed. Sequences with similarities > 97% considered as single OTU. We visualized Mothur output using Phyloseq package of R^41^. The significance of the differences in Alpha diversity indices i.e., ACE, Observed OTUs, Chao1, Shannon, Simpson and Inverse Simpson were estimated using microbial R package^42^. Top 50 OTUs were selected on the basis of abundance in the samples to visualize bacterial composition within the samples. The beta diversity estimations were performed through Principal Coordinate (PCA) and Cannonical Correlation Analysis (CCA) using weighted UniFrac distances^43^. We compared the fecal microbiome between macaque groups with different feeding behaviour (leftover feeding and crop-raiding) and geographical origin (Tawang and West Kameng) through Permutational Multivariate Analysis of Variance (PERMANOVA) test implemented in vegan R package using Bray–Curtis distance. The effect of feeding habits on gut microbiome was analysed between the crop-raiding and leftover feeding groups after exclusion of MT1398 from group A and MT2173 from group B as they showed dysbiosis. We identified differentially abundant taxa between the study groups using LeFSe test. Metagenomic function prediction was performed using Tax4Fun2 R package. Differentially abundant features were identified at level 2 and level 3 KEGG Orthology groups (KOs) through LEfSe analysis in Galaxy server of Huttenhower lab^44^. Metabolic pathways were compared between crop-raiding and leftover feeding groups and between dysbiotic (MT2173and MT1398) and non-dysbiotic (other samples).

### Ethics declarations

This was exempted as all samples were collected non-invasively without disturbing the macaques. Necessary permission was obtained from the State Forest Department Arunachal macaque.

## Results

### Species identification and assigning genealogical relationship

The obtained sequences clustered with known sequences of *M. munzala* in ML phylogenetic tree with high bootstrap support (Fig. S2). We observed that all analysed individuals were unrelated except two individuals- MT 2175 and MT2180, which were full-siblings and belonging to the group-B and group-C, respectively.

### Gut microbiome composition and alpha diversity

**Figure 2:**
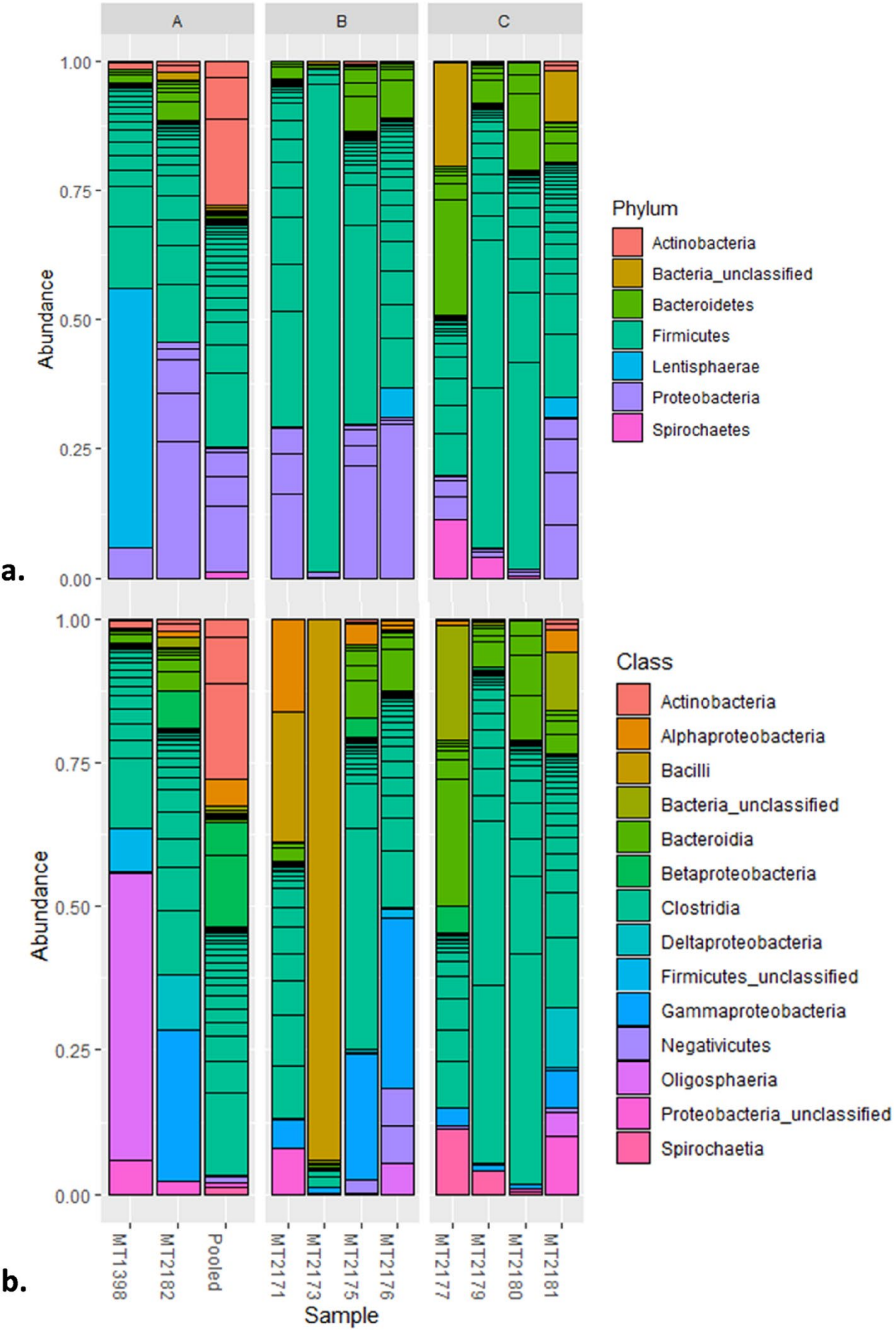
Gut microbiome composition in three groups of *M. munzala*. Phylum level (**a**) and class level comparison through relative abundance of bacterial taxa.

**Figure 3:**
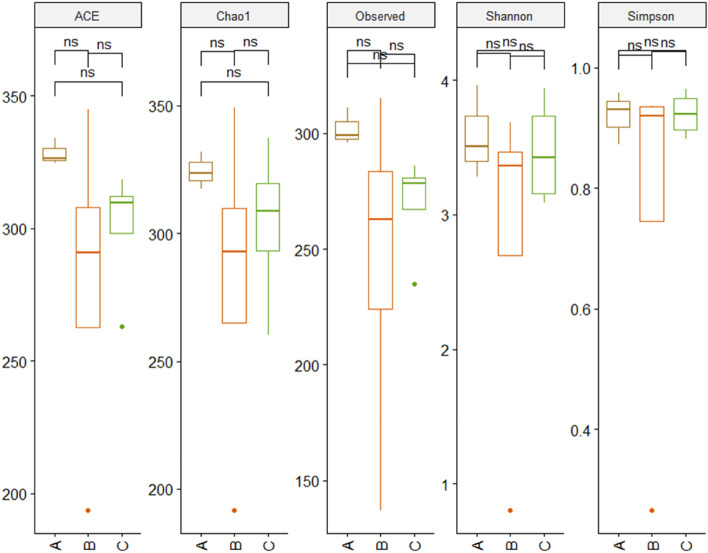
Comparison of different alpha diversity indices (ACE, Chao1, observed OTUs, Shannon and Simpson) between study groups shows no significant difference.

We obtained a total of 662 OTUs in 11 samples and the Firmicutes was the principal phylum followed by Proteobacteria and Bacteroidetes (Fig. 2). The Clostridia was found to be the dominant class in all samples except sample– ‘MT2173’and ‘MT1398’. The sample, ‘MT2173’ represented complete dominance (~ 95%) of the order Bacillales, contributed by a single OTU (OTU1) and showed 100% sequence similarity with genus *Solibacillus*. We did not find any significant difference between sample groups in any of the alpha diversity measures *i.e.* Observed OTUs, Shannon, Simpson, Chao1 and ACE (Fig. 3) while the ‘MT2173’ appeared as outlier in Shannon, Simpson, Chao1 and ACE indices. Another sample, ‘MT1398’ from group-A showed a lower level of dysbiosis with excess of Lentisphaerae.

### Beta diversity and differential abundance

**Figure 4:**
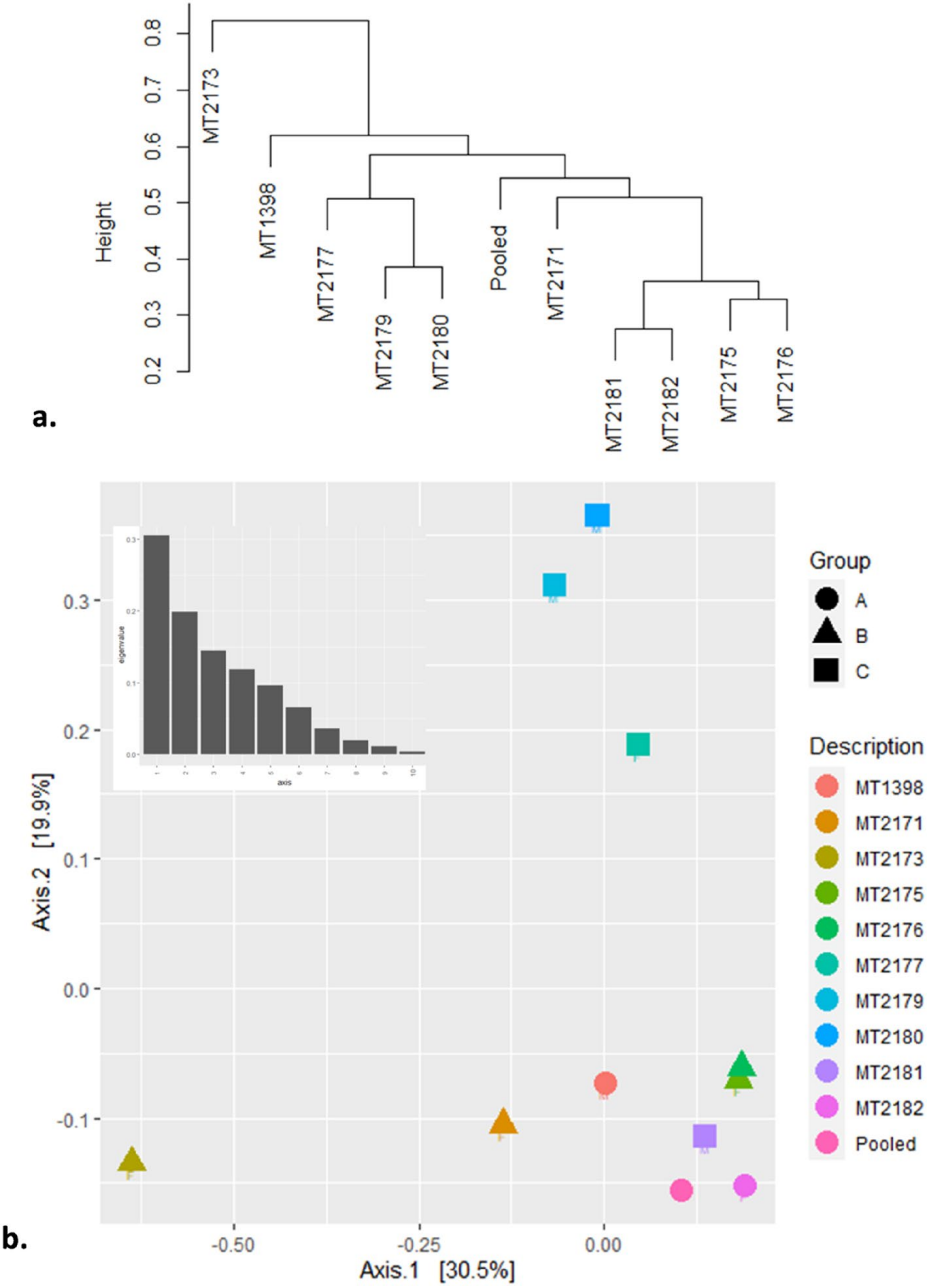
Beta diversity comparion between all samples. Cluster dendrogram (**a**) and PCoA plot (**b**) generated using weighted UniFrac distances.

**Figure 5:**
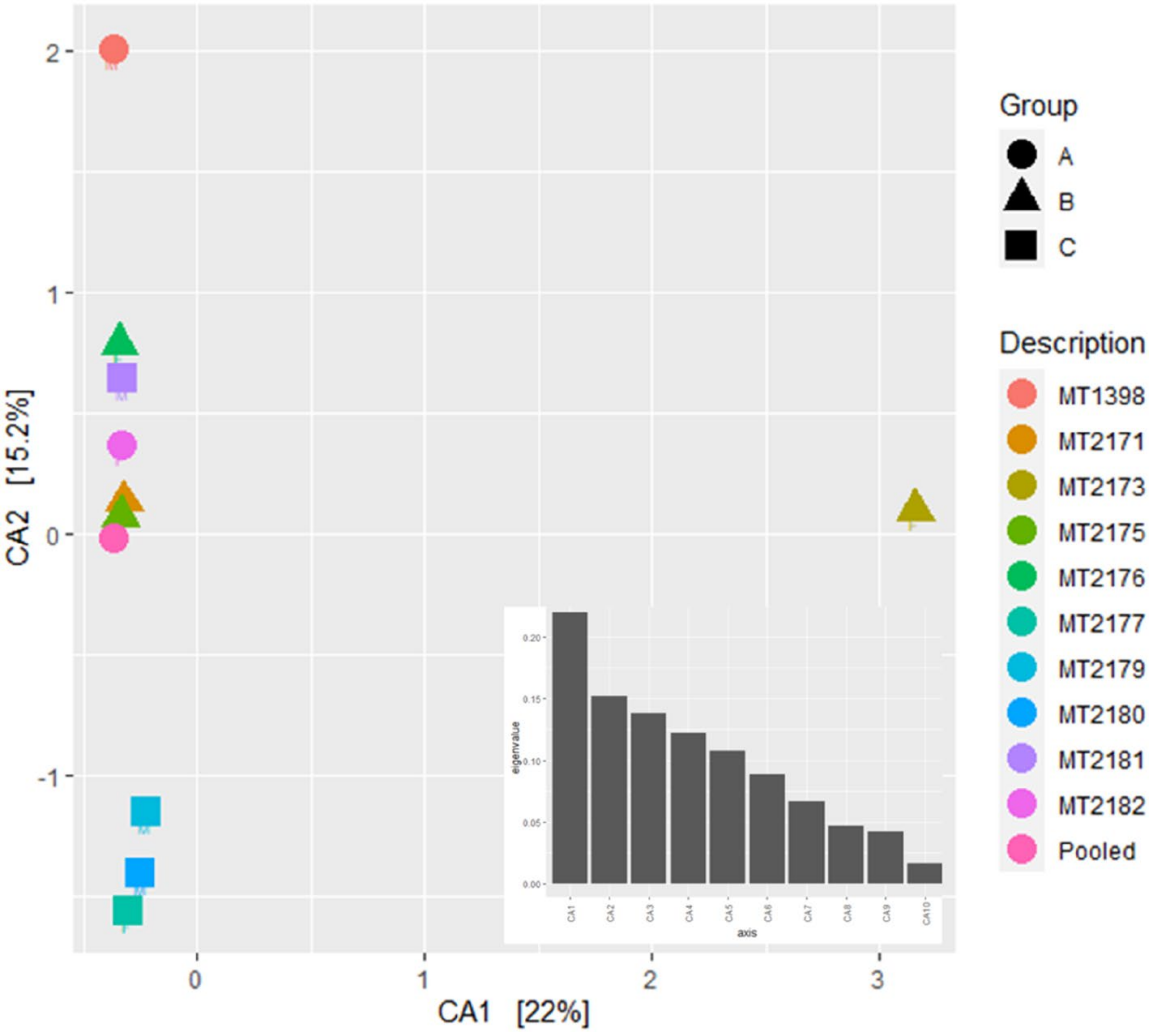
Cannonical correlation plot showing three out of four samples in group C clustered together and the dysbiotic sample (MT2173) showed highest variation from other samples.

**Figure 6:**
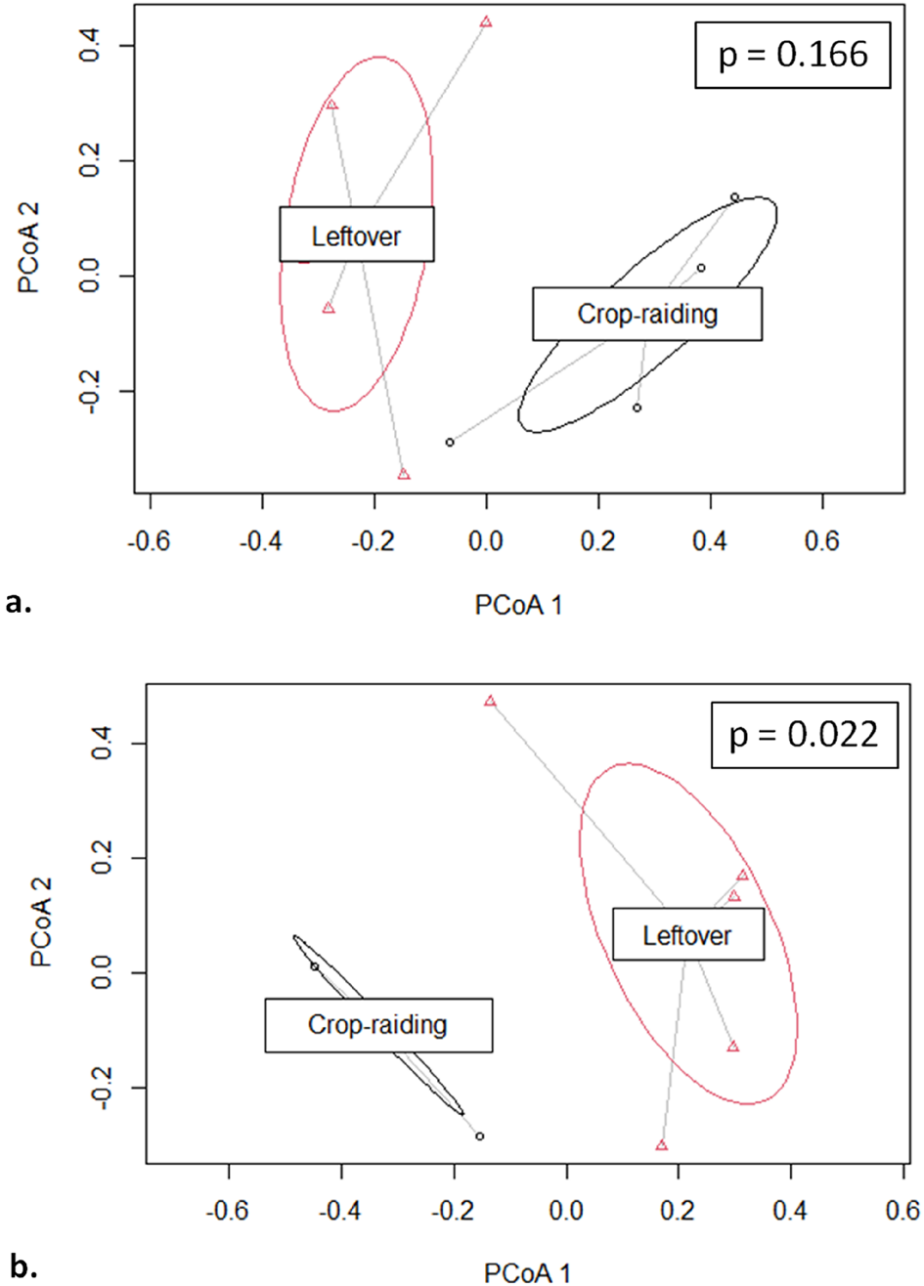
PCoA plot of Multivariate homogeneity of groups dispersions (variances) using Bray–Curtis distance. Multivariate dispersions in Leftover feeding and crop-raiding group excluding dysbiotic samples (**a**) and the same after excluding one more sample-MT2181 from crop raiding group (**b**). *p* values of PERMANOVA tests provided in insets.

**Figure 7:**
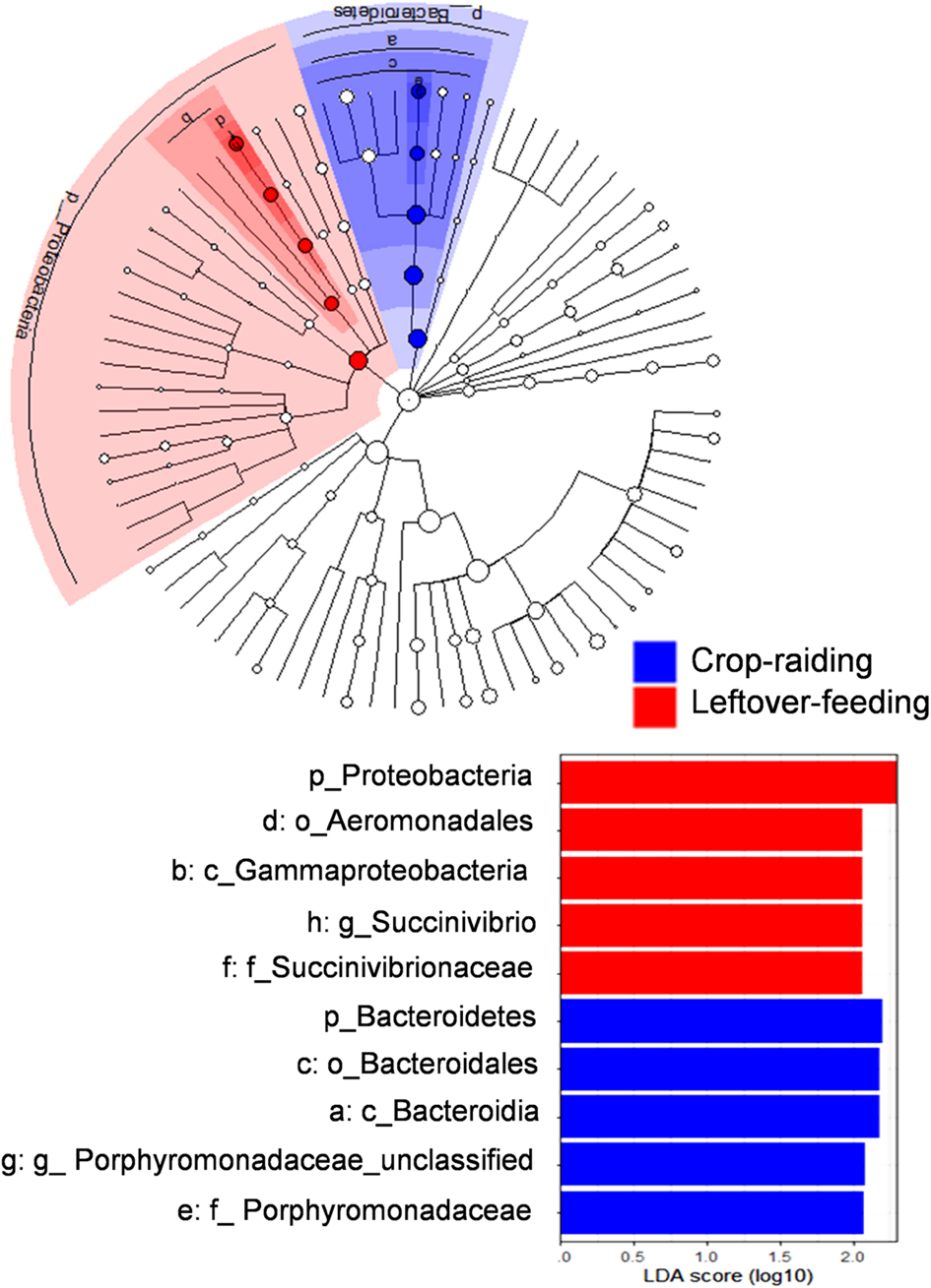
Differentially expressed bacterial taxa between crop-raiding and leftover-feeding groups found in LeFSe analysis (LDA > 2).

The cluster dendrogram using weighted UniFrac distance also showed that MT2173 had distinct composition of bacterial communities which is not related with other samples (Fig. 4a). The PCA using weighted UniFrac distance showed PC1-30.5%, PC2-19.9% and PC3-14.5% variation among the samples (Fig. 4b). The CCA showed CA1-22%, CA2-15.2% and CA3-13.8% variation among the samples and the CA1 segregated the sample MT2173 from remaining samples (Fig. 5). Furthermore, we found that three of the four samples belonging to group-C formed distinct clustered in PCoA, CCA and cluster dendrogram. However, upon PERMANOVA test, we did not observe significant difference between leftover-feeding (group A and B) and crop-raiding (group c) (*p* = 0.166: Fig. 6a). One sample-MT2181 from group C clustered with other samples of group A and B that assumed to feed on leftover despite belonging to crop raiding group (group C). Therefore, we performed PERMANOVA test again after removing the odd sample-MT2181. The results showed significant compositional difference between two groups with different feeding habit (p = 0.022: Fig. 6b). In contrast, we did not see geography has any role in shaping the gut microbiome (*p* = 0.475: Fig. S3). Further, we found that phylum Proteobacteria is enriched in leftover feeding group while phylum Bacteroidetes is differentially abundant in crop-raiding group (Fig. 7).

### Differentially expressed metabolic pathways

**Figure 8:**
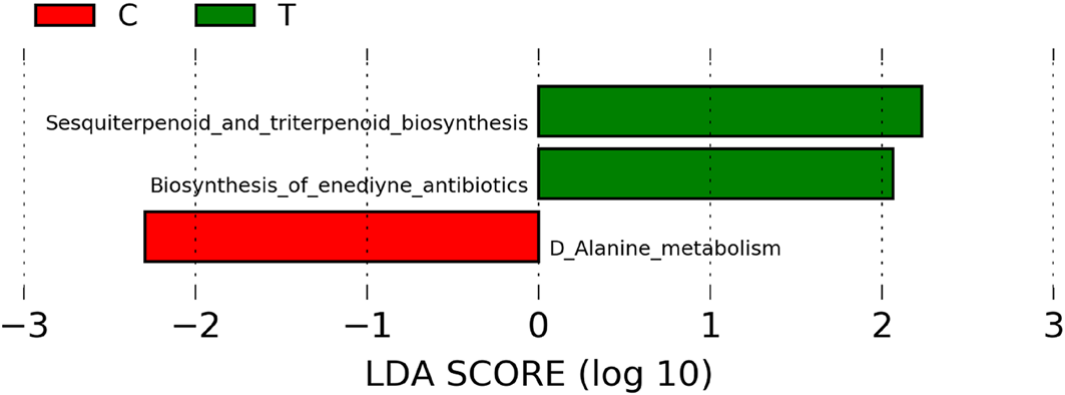
Differentially expressed metabolic pathways in normal (C) and dysbiotic (T) samples of *M. munzala *at level 2 (**a**) and level 3 (**b**).

We also did not observe differentially abundant features in level 2 KOs when groups were tested based on feeding habits and geography. On comparing leftover feeding group (group A & B) and crop raiding group (group C), we observed two differentially abundant features (Tropane, piperidine and pyridine alkaloid biosynthesis and novobiocin biosynthesis) of metabolic pathway enriched in crop-raiding group at level 3 KOs in LEfSe analysis (*p* < 0.05, LDA > 2). In contrast, on comparing the dysbiotic samples, MT2173 and MT1398 (considering test) to all other samples (considering control), we found enzymes of sesquiterpenoid and terpenoid biosynthesis and biosynthesis of enediyne antibiotics pathways were enriched in test group at level 3 KOs while amino D-Alanine metabolism was enriched in control group (Fig. 8: *p* < 0.05, LDA > 2).

## Discussion

During monitoring of macaques in field, we recorded that in Tawang, macaques often fed upon the leaves as well as on the human leftover food remains while macaques in West Kameng were observed to forage upon the cultivated crops as well as on the left over food. Here, we observed clustering of three out of four samples of the crop raiding group that indicates feeding habits is the principal driver for shaping the composition of the gut microbiome instead the geography. Further, the result from PERMANOVA test showed significant differences between crop-raiding group and leftover feeding group (*p* = 0.022) after exclusion of two dysbiotic (‘MT2173’ and ‘MT1398’) and one odd sample (MT2181). Thus, we found that feeding habits played a key role in shaping the gut microbiome in Arunachal macaque as revealed by the compositional shift in the gut microbiome between two groups. Further, we did not obtain any difference in the level 2 KO pathways possibly due to the limited characterization of bacterial species from environmental samples.

A few studies identified dysbiosis of gut microbiome with low alpha diversity in captive primates caused by increased stress, shift in diet and other external factors^2,16,17^ but dysbiosis in wild primates is not well reported. In the present study, we monitored an individual (MT2173) which had diarrhoea and seemed to be physically weak. Interestingly, this individual had distinct composition of bacterial community (complete dominance of *Solibacillus* sp.) and grouped distantly from others in cluster dendrogram. A previous study on rumen acidosis in dairy cows demonstrated positive correlation of *Solibacillus* with valerate, a branched chain volatile fatty acid [BCVFA]^45^, though the underlying mechanism behind the association is not clear yet. Interestingly, all the dysbiotic samples belong to leftover feeding group. This shift in diet prevails risk of various diseases which may become a threat for long term survival of this endangered macaque. Further, dysbiotic gut microbiota is reported to be harmful in several cases^46,47^ and so we raise our concern to the shift in the feeding habits of Arunachal macaques in Arunachal Pradesh. It is noteworthy that West Kameng district of Arunachal Pradesh possesses high human-macaque conflict due to crop-raiding by macaques and retaliatory killings of macaques by locals^23,48^. During the present study, we encountered group C macaques to forage upon the cultivated crops like cabbage, maize and tomato which plausibly shaped distinct gut microbial composition.

Notably, the macaque group (A & B) that feed upon the leftover food showed substantial fractions of shared bacterial taxa than the group C macaques having crop-raiding feeding habits. This is evident to prove that feeding habits have the principal role in shaping gut microbiome in Arunachal macaques. Further, the Tawang and West Kameng represent notable differences in the forest types. Though, Tawang has relatively large proportion of high elevation scrublands (4000–5250 m asl.), the habitats of Arunachal macaques in both districts comprise of temperate broadleaf (up to 3000 m) and mixed conifer and broadleaf forests (3000–4200 m)^48,49^. Monpa is the principal community in Tawang and they do not hunt due to their cultural beliefs^50^ but members of other tribes practice hunting^48^. While, a few forested habitats in Tawang are represented by community reserves where hunting is prohibited^51^. We observed macaques to retain an inherent behaviour of adaptation and they are often sighted nearby villages. Based on our field experience, we found their movements are largely governed towards the sites of ease availability of food resources. Thus, feeding habits have profound impact in shaping gut microbiome in Arunachal macaque over the geography. Since, we obtained significant differences in the bacterial taxa between macaques having different feeding habits, we also believe associating feeding behavior, nutrient characterization of the diet and the gut microbiome can provide valuable information in the context of linking bacterial taxa and with the various metabolic pathways or in digestion of the different types of food materials. The present study identified dysbiotic microbiome in *M. munzala* and provides empirical evidences for immediate conservation needs. Therefore, leftover-feeding may impose major threat by changing the natural diet of the macaques and increases the possibility of transfer of pathogen from human to macaques and vice-versa. Therefore, we propose that restoring the natural habitat of *M. munzala* by removal of waste, fencing the croplands and plantation of their natural food plants may help in conservation of this endangered macaque which in turn also minimize the level of human-macaque conflicts.

## Supplementary Information

Supplementary Information.
