# Supplementary material for: Linking gut microbiome with the feeding behavior of the Arunachal macaque (Macaca munzala)
Source: Sci Rep. 2021 Nov 9;11:21926. doi: 10.1038/s41598-021-01316-0 (PMC8578487; doi:10.1038/s41598-021-01316-0)
Supplement: Supplementary file 1 — Supplementary Information. [file 41598_2021_1316_MOESM1_ESM.docx]

Supplementary information

Table S1. Microsatellite genotypes, sex, genetic relatedness and grouping information of the individuals

| Individual | Loci | | | | | | | | | | | | | | | | | | Sex | Relatedness | Group |
| --- | --- | --- | --- | --- | --- | --- | --- | --- | --- | --- | --- | --- | --- | --- | --- | --- | --- | --- | --- | --- | --- |
|  | D01S548 | D01S548 | D05S1457 | D05S1457 | D06S1768 | D06S1768 | D06S501 | D06S501 | D08S1106 | D08S1106 | D19S255 | D19S255 | D22S685 | D22S685 | D04S2365 | D04S2365 | D13S765 | D13S765 |  |  |  |
| MT1398 | 198 | 198 | 128 | 128 | 198 | 206 | 0 | 0 | 135 | 135 | 151 | 151 | 348 | 348 | 290 | 294 | 213 | 213 | M | U | A |
| MT2171 | 206 | 206 | 136 | 140 | 214 | 226 | 170 | 178 | 147 | 151 | 119 | 119 | 236 | 236 | 290 | 302 | 221 | 233 | F | U | B |
| MT2173 | 206 | 206 | 132 | 132 | 222 | 226 | 170 | 170 | 131 | 147 | 119 | 119 | 236 | 328 | 294 | 294 | 233 | 233 | F | U | B |
| MT2175 | 198 | 206 | 132 | 132 | 214 | 218 | 170 | 186 | 139 | 147 | 119 | 119 | 236 | 236 | 282 | 282 | 221 | 221 | F | FS | B |
| MT2176 | 198 | 202 | 116 | 136 | 214 | 214 | 178 | 182 | 139 | 147 | 119 | 123 | 236 | 236 | 302 | 302 | 213 | 221 | F | U | B |
| MT2177 | 198 | 202 | 116 | 132 | 218 | 226 | 182 | 186 | 131 | 131 | 119 | 119 | 236 | 328 | 294 | 298 | 229 | 233 | F | U | C |
| MT2179 | 202 | 210 | 136 | 136 | 214 | 222 | 170 | 186 | 131 | 155 | 119 | 127 | 236 | 248 | 302 | 302 | 205 | 213 | M | U | C |
| MT2180 | 202 | 206 | 136 | 136 | 218 | 218 | 170 | 186 | 151 | 155 | 119 | 127 | 236 | 308 | 278 | 302 | 205 | 237 | M | FS | C |
| MT2181 | 202 | 206 | 132 | 136 | 222 | 218 | 178 | 186 | 147 | 147 | 119 | 119 | 324 | 324 | 286 | 290 | 205 | 229 | M | U | C |
| MT2182 | 198 | 206 | 124 | 136 | 222 | 246 | 182 | 186 | 151 | 155 | 119 | 119 | 236 | 328 | 282 | 282 | 217 | 233 | F | U | A |


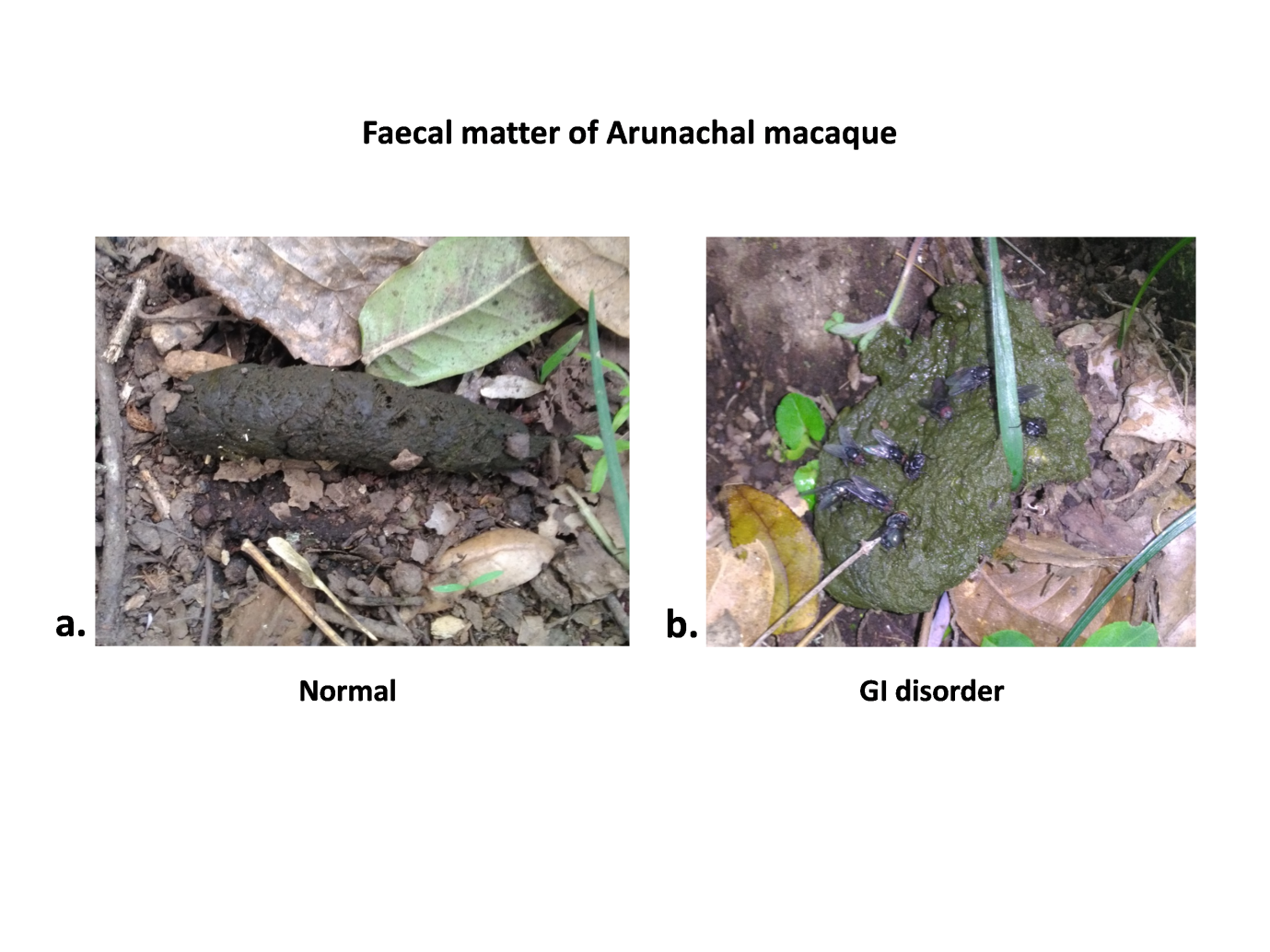


Figure S1. Faecal matters of *M. Munzala.* a. Normal faecal matter. b. Semisolid faecal matter (MT2173) indicating GI disorder.


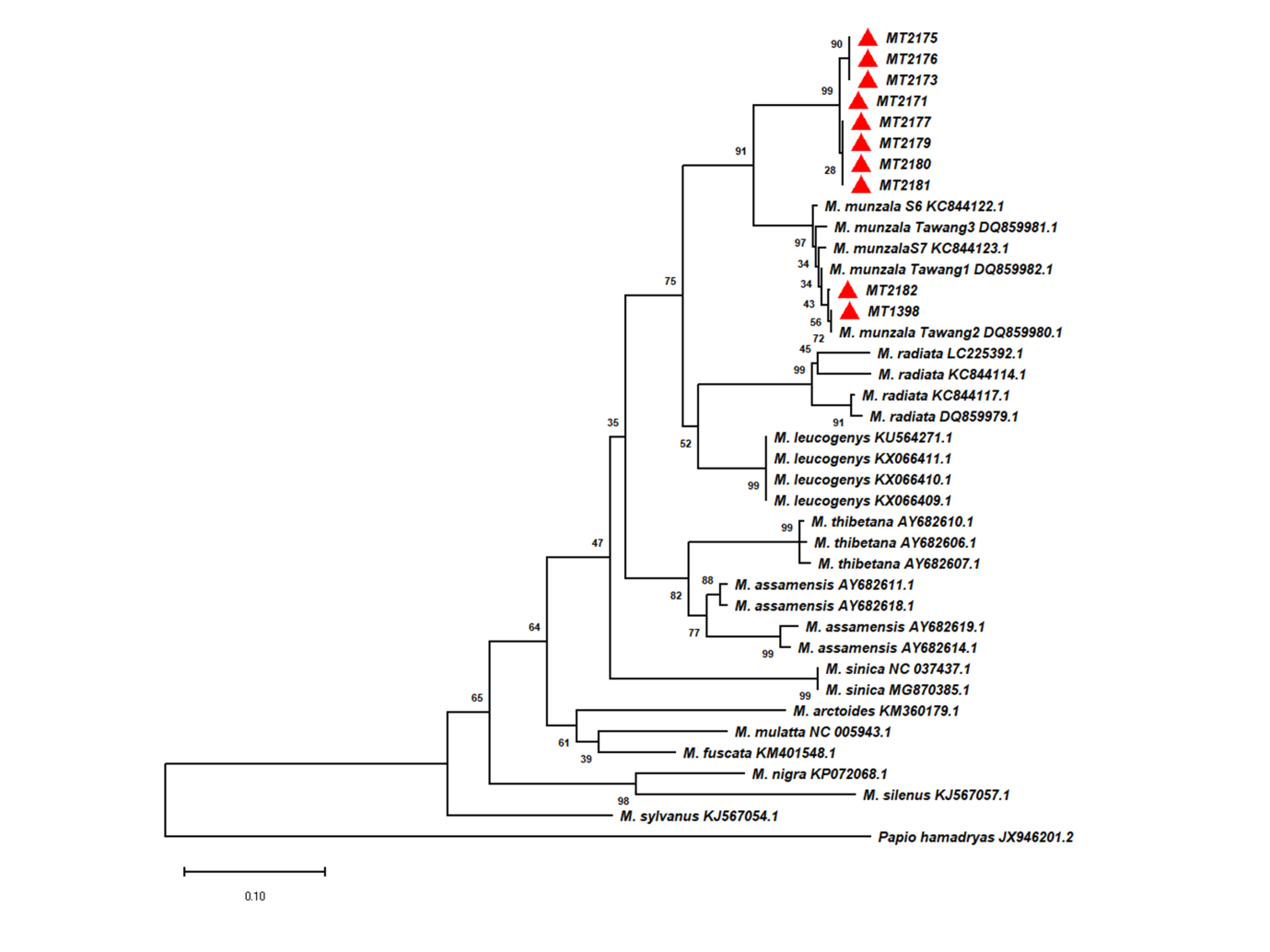
Figure S2. Maximum likelihood phylogenetic tree showing the relationship among macaques. The samples used in this study (marked with red triangle) clustered with known sequences of *M.munzala,* confirming the species of origin.


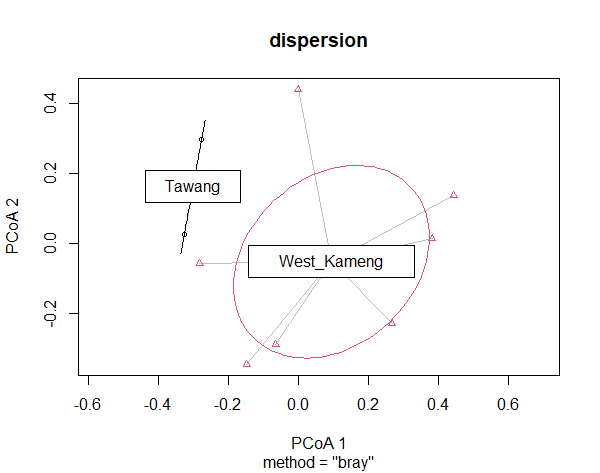


Figure S3. PCoA plot of Multivariate homogeneity of groups dispersions (variances) in two localities using Bray-Curtis distance (excluding dysbiotic samples). p=0.475.
